# Clinical Behavior of Aggressive Variants of Papillary Thyroid Carcinoma: A Retrospective Case–Control Study

**DOI:** 10.3390/cancers18020345

**Published:** 2026-01-22

**Authors:** Jovan Ilic, Nikola Slijepcevic, Katarina Tausanovic, Bozidar Odalovic, Goran Zoric, Marija Milinkovic, Branislav Rovcanin, Milan Jovanovic, Matija Buzejic, Duska Vucen, Boban Stepanovic, Sara Ivanis, Milan Parezanovic, Milan Marinkovic, Vladan Zivaljevic

**Affiliations:** 1 Clinic for Endocrine Surgery, University Clinical Center of Serbia, Pasterova 2, 11000 Belgrade, Serbia; katarinatausanovic@gmail.com (K.T.); odalovicb@gmail.com (B.O.); goranvanjazoric@gmail.com (G.Z.); rovcaninb@yahoo.com (B.R.); milanjovanovicceh@gmail.com (M.J.); matijabuzejic@gmail.com (M.B.); duskavucen@gmail.com (D.V.); bstepanovic93@gmail.com (B.S.); saraivaniss@gmail.com (S.I.); milan.parezanovic32@yahoo.com (M.P.); marinkovic.milan1995@gmail.com (M.M.); vladanzivaljevic@gmail.com (V.Z.); 2 Faculty of Medicine, University of Belgrade, 11000 Beograd, Serbia; 3 Faculty of Medicine, University of Pristina—Kosovska Mitrovica, 38220 Kosovska Mitrovica, Serbia; 4 Institute for Pathology, University Clinical Center of Serbia, 11000 Belgrade, Serbia; marija.milinkovic@yahoo.co.uk

**Keywords:** papillary thyroid carcinoma, aggressive variants, survival, clinicopathological characteristics, recurrence, risk stratification

## Abstract

Background/Objectives: Papillary thyroid carcinoma (PTC) is the most common endocrine malignancy. The classic variant (cPTC) is characterized by indolent behavior and excellent prognosis. However, rare subtypes of PTC most often exhibit adverse clinical behavior. The aim of the study was to assess the aggressiveness of rare variants of PTC by analyzing clinicopathological characteristics (CPCs) and survival outcomes. Methods: We analyzed 80 patients with rare PTC variants treated between 2009 and 2019 who were compared with cPTC and matched with a control group for age and tumor size. The variants were categorized into high-risk (HRV: tall cell, diffuse sclerosing, columnar cell, and hobnail variants), intermediate-risk (IRV: solid variant (SV)), and low-risk (LRV: oncocytic (OV) and Warthin-like (WLV)) variants. Different CPCs (capsule and blood vessel invasion, lymphonodal metastases, microscopic and macroscopic extrathyroid extension, multifocal and bilateral presentation) and survival outcomes—overall (OS), disease-specific (DSS), and disease-free survival (DFS) were compared. Results: HRVs exhibited significantly more aggressive CPCs and worse OS, DSS, and DFS compared to cPTC (*p* < 0.001). IRVs showed no significant difference in CPCs or survival outcomes compared to cPTC. LRVs showed excellent survival but were associated with several unfavorable CPCs. Multivariate analysis identified classification in HRVs as the only independent predictor of recurrence (*p* = 0.014). Conclusions: Tumors in the HRV group should retain their status as aggressive PTC variants due to unfavorable behavior and poorer prognosis. SVs, despite earlier assumptions, do not exhibit aggressive characteristics. Although the OV and WLV have similar survival to cPTC, their potential for adverse CPCs requires caution.

## Simple Summary

Papillary thyroid carcinoma is one of the most indolent malignancies in the human population. However, there are several rare subtypes of this cancer, which are characterized by higher rates of recurrence, metastases, and death. Not all of these subtypes act with the same level of aggression. This is why the aim of this research is to investigate the aggressiveness profile of each of the subtypes, especially with regard to some newer information in the literature about them. In doing so, we could stipulate which subtypes and tumor characteristics would require additional caution and treatment steps in everyday practice.

## 1. Introduction

Papillary thyroid carcinoma (PTC) is the most common malignant endocrine tumor and accounts for approximately 84–85% of all thyroid gland malignancies [1,2]. The classical variant of PTC (cPTC) is well-differentiated and has an indolent clinical course with a five-year overall survival rate of 97.5%, and up to a 97% 10-year cancer-specific survival rate [2,3]. However, uncommon, rare forms of PTC are usually more aggressive, often distinguished by a higher degree of recurrence, local and vascular invasion, lymph node (LN) and distal metastases, and in some cases the lack of avidity to radioiodine (RAI) therapy, often resulting in a lower survival rate. These aggressive forms are the tall cell variant (TCV), diffuse sclerosing variant (DSV), columnar cell variant (CCV), hobnail variant (HV), and solid variant (SV) [2].

The aggressiveness of the SV has been a matter of discussion in recent years, especially after the introduction of the fifth version of the WHO histological classification of thyroid neoplasms [4,5,6]. Also, other rare forms of PTC have been studied during the last few decades such as the oncocytic variant (OV) and its own subtype, the Warthin-like variant (WLV) [7,8].

Most evidence shows that the OV and WLV do not exhibit a significantly more aggressive behavior regarding clinicopathological characteristics compared to cPTC, and that they have similar or marginally higher recurrence and survival rates [9,10,11,12].

The aim of this study was to investigate and evaluate the aggressiveness profile of all the aforementioned forms of PTC, especially regarding their unfavorable clinicopathological characteristics and survival rates. We compared cPTC to forms proven to be more aggressive in the literature (TCV, DSV, CCV, HV), as well as to those which are a matter of debate (SV) and to those considered to be as aggressive as cPTC (OV and WLV).

## 2. Materials and Methods

This was a retrospective, case–control study that included patients who underwent surgical treatment for thyroid follicular-cell carcinomas at a tertiary referral academic hospital, between January 2009 and January 2019. From this group of patients, we selected and analyzed data of all patients with rare forms of PTC and compared them to the control group which was formed out of patients with cPTC. All relevant demographic, clinical and pathohistological (PH) information was obtained from a prospectively maintained institutional database designed by our team of endocrine surgeons and developed by E-Smart Systems Ltd. in 2008 or by telephone contact with the patients or members of patients’ families, in the case of deceased patients. All consecutive eligible patients were included in the study. The study was conducted according to the guidelines of the Declaration of Helsinki, and approved by the Ethics Committee of the University Clinical Center of Serbia (No. 1600/49). An obligatory written informed consent was obtained from all the patients before the operative procedure to research the data from their medical histories and publish the scientific research, while preserving their anonymity.

All available histological slides of tumors classified as rare variants were retrospectively re-reviewed in 2025 by an experienced endocrine pathologist (M.M.) using the diagnostic criteria of the WHO 2022 (5th edition) classification of thyroid tumors. This re-review was performed to harmonize historical diagnoses with contemporary classification standards. We have used the following criteria during the review for the diagnosis of rare variants: For the TCV, more than 30% of tall cells present in the tumor; for the DSV, 100% diffuse unilateral or bilateral involvement, without dominant tumor mass; for the CCV, observed presence of columnar cells with pale eosinophilic cytoplasm and prominent pseudo-stratification; for the HV, more than 30% of tumor having hobnail cells; and, for the SV, more than 50% of solid growth pattern of the primary tumor, nuclei appearance that matches the appearance of the nuclei of classic variant papillary carcinoma, and the absence of tumor necrosis. For the OV, the histological criteria entailed well-developed papillae lined with oncocytic cells, and, for the WLV, the presence of papillae lined with oncocytic cells, but with papillary core containing lymphoplasmacytic infiltrate.

In addition to the aforementioned pathohistological criteria, the inclusion criteria were as follows: (1) patient age ≥ 18 years; (2) no history of prior thyroid surgery; and (3) documented cause of death confirmed either by family members or verified clinical status at the last follow-up, which occurred at least one year prior to the start of the study.

Patients were excluded if they met any of the following criteria: (1) pathohistological reports indicating a predominantly classic or follicular variant of papillary thyroid carcinoma (PTC) with only a minor component or focal presence of a rare variant; (2) age < 18 years; or (3) incomplete essential clinical data or a time from last follow-up exceeding one year prior to the study onset.

All patients with rare variants who met the inclusion criteria were grouped into one of the three formed categories, according to the historical data based on clinicopathological behavior and survival: (1) high-risk variants (HRVs)—TCV, DSV, CCV, HV, (2) intermediate-risk variant (IRVs)—thought to be aggressive but with recently conflicting evidence, especially regarding mortality rate—SV and (3) low-risk variants (LRVs), believed to be as aggressive as cPTC—OV and WLV.

Each of the rare variants were individually matched to a classical (papillary) form of PTC (cPTC) for age (younger or older than 55 years) and tumor size (1–20 mm, 21–40 mm, 41–60 mm, >60 mm). This way, the control group was double the size of the largest examined group (IRV) and more than 5 times the size of the smallest examined group (HRV).

### Statistical Analysis

Each of the three groups (HRV, IRV, and LRV) were compared to the control group for differences in the distribution of clinicopathological variables—capsular invasion, vascular invasion, microscopic and gross extrathyroidal extension, multifocal and bilateral presentation, LN metastases, and the use of RAI therapy using the McNemar method for matched pairs—if more than 20% of the cells in the contingency table had an expected count of less than 5, the Fisher test was used. Overall survival (OS) and disease-specific survival (DSS) were calculated from the surgical excision date of the primary tumor to the date of death (depending on the cause) or last check-up. The disease-free survival (DFS) was calculated from the date of surgical excision of the primary tumor to the date of the first relapse or last follow-up. Survival curves were plotted using the Kaplan–Meier method, and the statistical comparisons were performed using the log-rank test. Univariate and multivariate regression analysis were performed using the Cox regression model. All statistical tests were two-sided. A *p*-value < 0.05 was considered statistically significant, and a *p*-value < 0.01 was considered highly statistically significant. The results are shown as tables and graphs. Data is shown as n (%), arithmetic mean ± standard deviation. All data was processed in the IBM SPSS Statistics version 25 (SPSS Inc., Chicago, IL, USA) software package.

## 3. Results

Of the total of 1867 patients treated for thyroid follicular-cell carcinomas during the ten-year period, 80 had confirmed a PH report of rare variants of PTC—7 cases of the TCV (8.8%), 5 of the DSV (6.3%), 2 of the CCV (2.5%), 1 of the HV (1.3%), 40 patients had the SV (50%), 14 had the OV (17.5%), and 11 patients had the WLV (13.8%). On retrospective histopathological re-review using WHO 2022 criteria, none of the included cases fulfilled the diagnostic requirements for differentiated high-grade thyroid carcinoma. Among the patients with rare variants of PTC, 19 (23.8%) were male and 61 (76.3%) were female, with an average age of 51.7 ± 15.9 (from 18 to 86) (Table 1). The incidence of all rare variants in our series was 4.3%.

**Table 1:** Patient and tumor characteristics.

Characteristics	Value (%)
**Number of patients**	80
**Rare variant pathology**	
Tall cell	7 (8.8%)
Diffuse sclerosing	5 (6.3%)
Columnar cell	2 (2.5%)
Hobnail	1 (1.3%)
Solid	40 (50%)
Oncocytic	14 (17.5%)
Warthin-like	11 (13.8%)
**Gender**	
Male	19 (23.8%)
Female	61 (76.3%)
**Age, years** (mean ± sd)	51.7 ± 15.9
**FNAB**	
Bethesda I	0
Bethesda II	24 (30%)
Bethesda III/IV	23 (28.7%)
Bethesda V	5 (6.3%)
Bethesda VI	7 (8.8%)
Not performed	21 (26.3%)
**Procedure**	
Total thyroidectomy	48 (60%)
Staged thyroidectomy	4 (5%)
Near-total thyroidectomy	6 (7.5%)
Thyroid lobectomy	13 (16.3%)
Thyroidectomy with neck dissection	9 (11.3%)
**Primary tumor size, mm** (mean ± sd)	32.38 ± 30.42
**Clinicopathological characteristics**	
Capsular invasion	31 (38.8%)
Vascular invasion	17 (21.3%)
LN metastases	14 (17.5%)
Microscopic ETE	16 (20%)
Gross ETE	9 (11.3%)
Multifocal presentation	31 (38.8%)
Bilateral presentation	26 (32.5%)
Tumor recurrence	8 (10%)
RAI treatment	31 (38.8%)
Deaths	6 (7.5%)
Cancer-specific deaths	4 (5%)

LN—lymphonodal; ETE—extrathyroid extension; RAI—radioactive iodine.

Fine needle aspiration biopsy (FNAB) was performed on 59 (77.5%) patients. In 24 (30%) patients, the FNAB indicated benign thyroid conditions, and 23 (28.7%) patients were suspected of having a follicular or oncocytic neoplasm. PTC was highly suspected in five (6.3%) patients, and in seven (8.8%) patients, PTC was confirmed on biopsy (Table 1).

Initial total thyroidectomy was performed in 48 (60%) patients, staged thyroidectomy in 4 (5%) patients, near-total thyroidectomy in 6 (7.5%) patients, thyroid lobectomy in 13 (16.3%) patients, and total thyroidectomy with neck dissection in 9 (11.3%) patients (Table 1). The primary tumor size in all cases ranged from 6 to 240 mm, with an average of 32.38 ± 30.42 mm, of which 58.7% were over 2 cm in size.

Capsular invasion was observed in 31 (38.8%), vascular invasion in 17 (21.3%), and LN metastases in 14 (17.5%) patients with a rare variant of PTC. Microscopic ETE was noted in 16 (20%) patients and gross ETE in 9 (11.3%). A total of 31 patients (38.8%) had a multifocal tumor presentation, and 26 patients (38.8%—corrected for the patients who underwent thyroid lobectomy from total percentage) had a bilateral tumor presentation in both thyroid lobes. Eight patients (10%) had tumor recurrence. Radioactive iodine (RAI) treatment was given postoperatively to 31 patients (38.8%), to most patients only once, twice to six patients, and three times to two patients. During the follow-up, death was observed in six patients (7.5%)—four were cancer-specific due to thyroid carcinoma.

### 3.1. Clinicopathological Features Among the Four Subgroups

Comparing the clinicopathological features of tumors in three formed variant subgroups (HRV, IRV, LRV) to the control group (cPTC), a high statistical significance (*p* < 0.001) was observed in the incidence of almost all unfavorable clinicopathological characteristics in the HRV group compared to cPTC, except for capsular invasion, which was only statistically significant (*p* < 0.05). IRVs did not show significantly more unfavorable clinicopathological characteristics compared to cPTC and even had a highly significant (*p* < 0.001) lower risk of having bilateral presentation. The LRV group had a highly significant (*p* < 0.001) risk of forming vascular invasion and multifocal and bilateral presentation, and a significant risk (*p* < 0.05) of presenting with capsular invasion and LN metastases, as well as higher chances of being treated with RAI compared to cPTC. There were no distant metastases observed across either of the cases subgroups or the control group (Table 2).

### 3.2. Survival Analysis for the Four Subgroups

**Figure 1:**
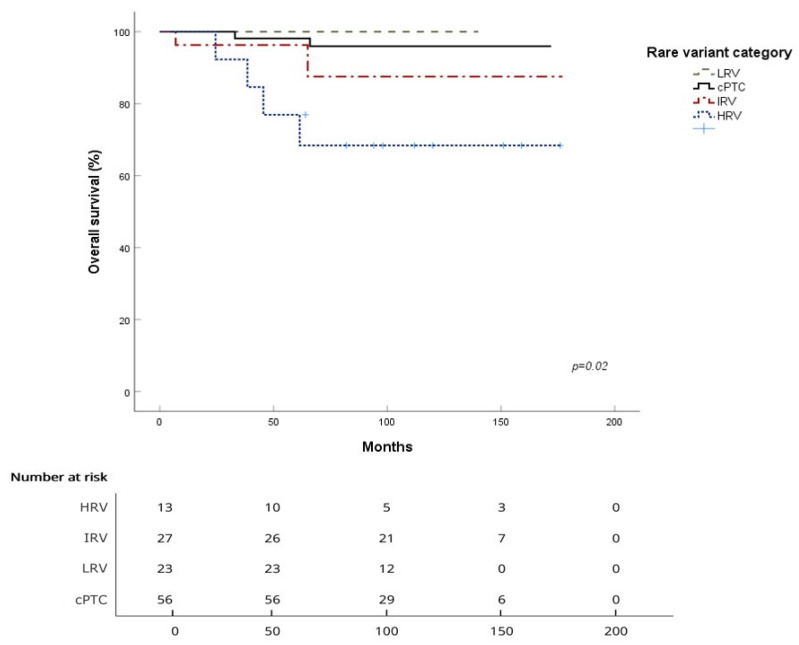
Overall survival curves for each of the four subgroups with number at risk at each time point (HRV—high-risk variant; IRV—intermediate-risk variant; LRV—low-risk variant; and cPTC—classical variant).

For overall survival (OS), the mean survival in months was 167.8 ± 2.9 for cPTC, 136.4 ± 16.5 for HRV, 165.6 ± 7.6 for IRV, and could not be calculated for LRV. The 5-year OS rates were 98.1% for cPTC, 76.9% for HRV, 96.3% for IRV, and 100% for LRV, while the 10-year OS rates were 96.0%, 68.4%, 87.5%, and 100%, respectively. The log-rank test revealed a significant difference between cPTC and HRV (*p* = 0.001), but no significant differences between cPTC and IRV or LRV (*p* = 0.58 and *p* = 0.36, respectively) (Table 3, Figure 1).

**Figure 2:**
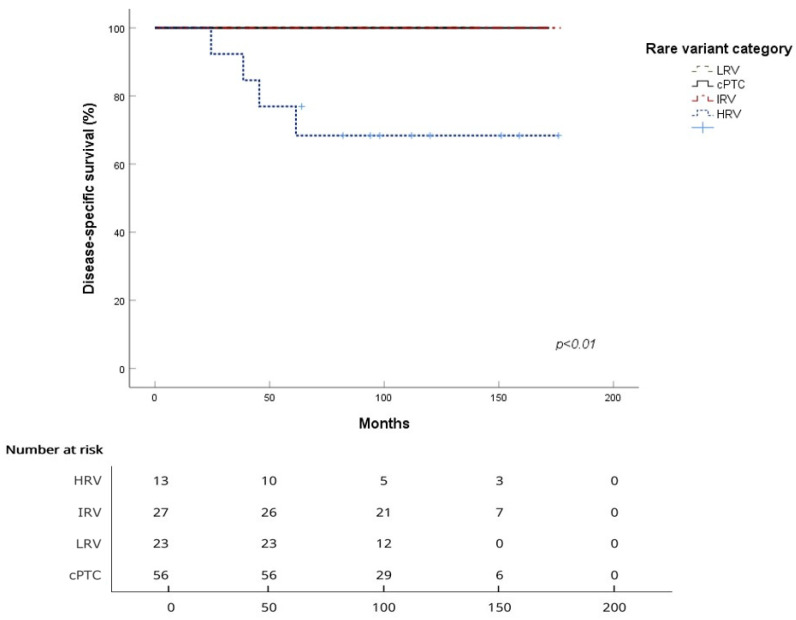
Disease-specific survival curves for each of the four subgroups with number at risk at each time point (HRV—high-risk variant; IRV—intermediate-risk variant; LRV—low-risk variant; and cPTC—classical variant).

For disease-specific survival (DSS), the mean survival in months for HRV was 136.4 ± 16.5 months, and no cancer-specific deaths were observed in cPTC, IRV, and LRV. The 5-year DSS rates were 100% for cPTC, 76.9% for HRV, and 100% for both IRV and LRV, while the 10-year DSS rates were 100% for cPTC, 68.4% for HRV, and stayed at 100% for IRV and LRV. A log-rank test indicated a significant difference between cPTC and HRV (*p* < 0.001), but no other comparisons yielded significant results (Table 3, Figure 2).

**Figure 3:**
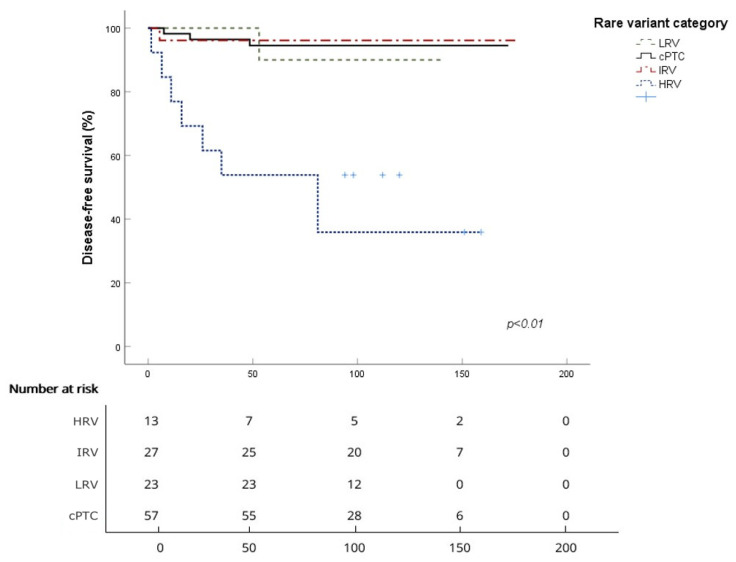
Disease-free survival curves for each of the four subgroups with number at risk at each time point (HRV—high-risk variant; IRV—intermediate-risk variant; LRV—low-risk variant; and cPTC—classical variant).

For disease-free survival (DFS), the mean survival time in months was 164.6 ± 4.2 for cPTC, 89.2 ± 18.7 for HRV, 170.5 ± 6.4 for IRV, and 136.3 ± 3.5 for LRV. The 5-year DFS rates were 96.4% for cPTC, 53.8% for HRV, 96.2% for IRV, and 100% for LRV, while the 10-year DFS rates were 94.5%, 40.4%, 96.2%, and 90%, respectively. The log-rank test showed a significant difference between cPTC and HRV (*p* < 0.001), while comparisons with IRV and LRV did not reveal significant differences (*p* = 0.8 and *p* = 0.84, respectively) (Table 3, Figure 3).

**Table 3:** Survival rate and times among the four subgroups.

Survival	cPTC	HRV	IRV	LRV
Mean OS (months)	167.8 ± 2.9	136.4 ± 16.5	165.6 ± 7.6	/
5-year OS	98.1%	76.9%	96.3%	100%
10-year OS	96.0%	68.4%	87.5%	100%
Log-rank (vs. cPTC)		***p* = 0.001**	*p* = 0.58	*p* = 0.36
Mean DSS (months)	/	136.4 ± 16.5	/	/
5-year DSS	100%	76.9%	100%	100%
10-year DSS	100%	68.4%	100%	100%
Log-rank (vs. cPTC)		***p* < 0.001**	*/*	*/*
Mean DFS (months)	164.6 ± 4.2	89.2 ± 18.7	170.5 ± 6.4	136.3 ± 3.5
5-year DFS	96.4%	53.8%	96.2%	100%
10-year DFS	94.5%	40.4%	96.2%	90%
Log-rank (vs. cPTC)		***p* < 0.001**	*p* = 0.8	*p* = 0.84

*p*-value was bolded if statistically significant. HRV—high-risk variant; IRV—intermediate-risk variant; LRV—low-risk variant; cPTC—classical variant; OS—overall survival; DSS—disease-specific survival; DFS—disease-free survival.

These findings underscore the differences in survival outcomes between patient groups, with HRV showing less favorable outcomes in terms of OS and DFS compared to cPTC, while the IRV and LRV groups had comparable outcomes to cPTC.

When considering factors that could affect DFS, the univariate analysis showed seven factors that could be associated with poorer prognosis, i.e., higher recurrence rate—vascular invasion, multifocal presentation, bilateral presentation, LN metastases, and microscopic and gross ETE, as well as classification of tumor in the HRV subgroup. In multivariate analysis, classification within the high-risk variant (HRV) group remained significantly associated with disease recurrence (*p* = 0.014), within the limitations of a retrospective cohort and a limited number of recurrence events (Table 4).

## 4. Discussion

The aim of the study was to investigate the aggressiveness profile and survival rates of rare variants of PTC by grouping them into three groups based on historical research evidence. Following that, we compared each group to cPTC to establish which of the rare variants had an unfavorable clinicopathological profile and gave an explanation on what may have influenced their aggressiveness over the years.

Rare variants of PTC collectively account for a small proportion of all PTC cases. Large cohort studies report that rare variants represent approximately 1.1% of PTC cases, with the most common among them being the TCV, followed by the SV, and the rarest of them being the CCV [13,14]. The incidence of rare variants in our study was 4.3%, which is higher than the average incidence of 1.1% which was reported in the multicentric study of Kim et al., but comparable to the incidence of some of the nine institutions that were included in their study, which ranged from 0% to 5.6% [14]. The most common variant in our cohort was the SV, followed by the OV and WLV, then the TCV, the DSV, and finally the CCV and HV being the rarest.

The larger incidence in our study might also be in part due to the rising incidence of rare PTC variants, with an annual percentage increase of 9.1% for aggressive subtypes compared to 5.1% for cPTC or the follicular variant of PTC. In the retrospective cohort study conducted by Ho et al., who established this trend, the aggressive subtype category also included poorly differentiated thyroid carcinoma and the insular variant of PTC [15].

In earlier decades, the pathohistological reports were less detailed than today, and many PTCs were classified just as PTC without a detailed description of aggressive features and subtypes. Historically, PTC classification was less detailed or it has evolved over time, including how certain subtypes/aggressive features were not always differentiated in older reports. Also, not all world regions report rare subtypes in the same way, mainly due to differences in the number of specialized, tertiary institutes in certain regions [16,17,18].

FNAB is considered to be a leading, minimally invasive diagnostic tool for diagnosing thyroid malignancies preoperatively [19]. It is the only viable method for differentiating benign thyroid nodules from malignant ones, with a sensitivity of 80.48% and a specificity of 83.33% on its own, and with the potential to reach 100% specificity if combined with the ultrasonographic classification system of thyroid nodules (TI RADS score) [20]. There have been reports of very low sensitivity (11.5%), but with a reliable, 100% specificity, and caution is advised when interpreting false negative results of intermediate FNAB categories [21,22]. Examining FNAB results in our cohort, which exclusively had malignancies, 30% had a Bethesda II result, which significantly impacted the sensitivity. Diagnosing rare PTC subvariants by FNAB can be challenging due to overlapping cytological and nuclear features with cPTC. Experienced pathologists can sometimes differentiate between the subvariants based on cytological findings [23,24,25].

### 4.1. High-Risk Variants

In this study, variants with clear evidence of aggressiveness throughout the literature were grouped in the “high-risk variants” (HRVs) group. This group included the TCV, DSV, CCV, and HV.

**Figure 4:**
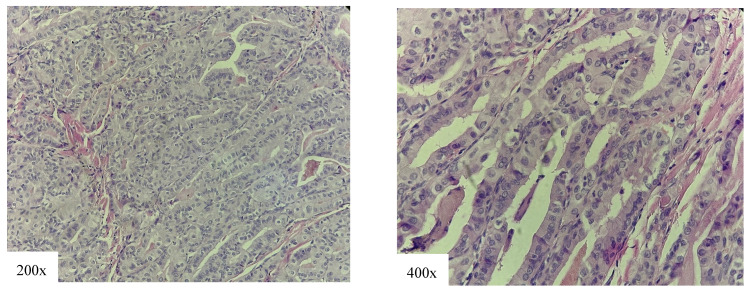
Tall cell variant of papillary thyroid carcinoma: densely packed papillae and follicules, tumor cells 3 times longer than wider, eosinophilic cytoplasm with intranuclear inclusions (nuclear features of papillary thyroid carcinoma).

The TCV (Figure 4) represents one of the most common aggressive subtypes of PTC. According to most authors, the percentage of tall cells that should be present for histological diagnosis is 30% [26,27], which is a criterion our team was led by. The TCV is more frequent in women, with the average age of patients being higher than that of patients with cPTC [28,29,30]. Recently, a significant rise in incidence of this subtype has been reported [31].

**Figure 5:**
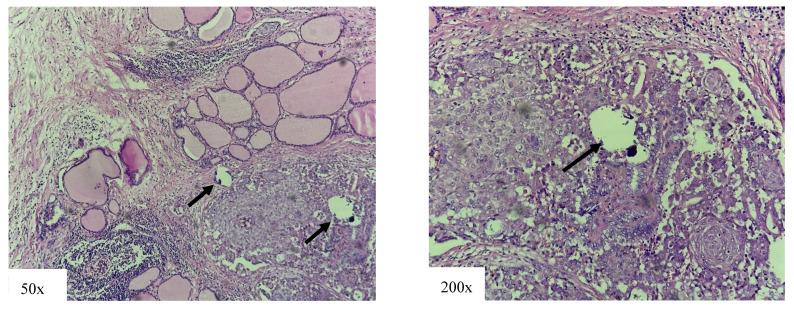
Diffuse sclerosing variant of papillary thyroid carcinoma: multifocal tumor with tumor cells arranged in solid cell nests with presence of squamous metaplasia and parts of psammoma bodies (arrow). Chronic lymphocitic thyroiditis is also present.

Around 6% of PTC cases are DSVs (Figure 5) [26,32]. This variant is most commonly found in younger female patients [32,33,34,35], with a high incidence in the pediatric population [36,37].

**Figure 6:**
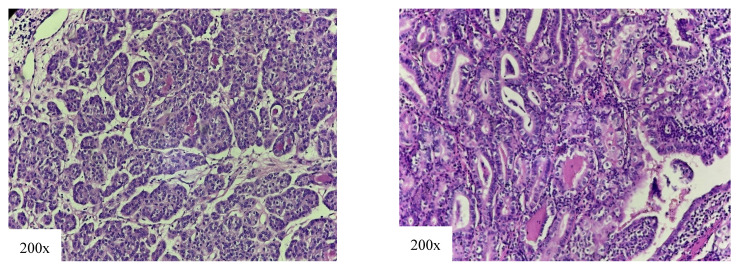
Columnar cell variant of papillary thyroid carcinoma: cells arranged in tightly packed columns with eosinophilic cytoplasm and pseudostratification.

One of the rarest subtypes is the CCV, accounting for around 0.1–0.2% of PTC (Figure 6) [38,39]. The average age at diagnosis is similar to cPTC, spanning from 34 to 57 years, with a slight predomination of female cases [40,41,42]. Evidence suggests poorer response to RAI therapy in this subtype of PTC [13].

**Figure 7:**
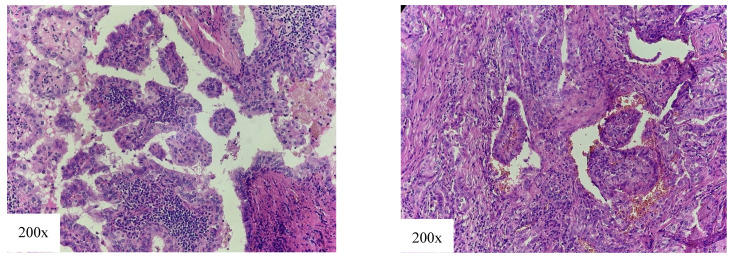
Hobnail variant of papillary thyroid carcinoma: papillary growth pattern, tumor cells with onocytic cytoplasm, and enlarged nuclei bulging from the apex of papillae.

The HV presents one of the most recently described forms of PTC, with the first histological features described in 2004 [43]. The diagnostic criteria are mostly dependent on the loss of cellular polarity (elevated nuclear position) in ≥30% of cells, with the exclusion of other subtypes of PTC [44], which is the criterion we follow for pathohistological diagnosis (Figure 7).

Most of the studies that analyzed these four subvariants have been presented as single and multicentric studies or meta-analyses which compared each individual variant to cPTC. All of these studies and their presented evidence strongly emphasize the aggressiveness of the HRVs with little to no degree of contradiction.

In our study, we had seven TCVs, five DSVs, two CCVs, and only one HV. Naturally, because of the small number of cases, we unified them in a single HRV subgroup, as examination of each individual group would be statistically implausible.

Capsular invasion was significantly more frequent in HRVs (60%) compared to cPTC (26.3%, *p* = 0.015), and the rate of vascular invasion in HRVs was 53.3%, significantly higher than in cPTC (5%, *p* < 0.001). Longheu et al. reported a high statistical significance for an increased rate of angiolymphatic invasion (17.14% vs. 5.06%, *p* = 0.01) [45]. Liu et al.’s meta-analysis showed significantly higher odds of vascular invasion in the TCV (odds ratio (OR) = 2.12; 95% CI: 1.50–3.00, *p* < 0.001) and Vuong et al. reported similar findings in the DSV (OR = 5.33; 95% CI: 3.08–9.23) [33,46]. Donaldson et al. noted both capsular and vascular invasion in over 60% of HV cases [43].

LN metastases were present in 40% of HRV cases compared to 5% of cPTC (*p* < 0.001). In studies published by Longheu and Axelsson, the TCV has shown significantly higher LN metastasis rates—between 45.7% and 51% (vs. between 13.29% and 22% for cPTC, *p* < 0.0001) [29,45]. A meta-analysis by Liu et al. showed that the OR for LN metastases in the TCV was 1.85 (95% CI = 1.54–2.24, *p* < 0.001) and the DSV has even higher odds of an LN metastasis rate (OR = 2.91, 95% CI = 1.88–4.50 and OR = 5.40; 95% CI: 2.82–10.35) [33,46,47]. The LN involvement was found in 66–68% of HV cases [43,48]. Although evidence on the CCV is limited, studies like Song et al. identified LN involvement as a poor prognostic indicator in the CCV, as well [49].

Our study did not identify any cases of distant metastases in either the HRV or cPTC groups. This finding contrasts with the existing literature, which consistently reports higher rates of distant spread in high-risk variants. The TCV has shown distant metastases in 5.7–14% of cases and a meta-analysis reports a significantly elevated risk compared to cPTC (OR = 3.10, 95% CI = 1.61–5.98, *p* < 0.001) [29,45,46]. The DSV similarly shows increased incidence, with reported odds ratios ranging from 2.2 to 3.6 [33,47]. The HV demonstrates particularly high rates, with metastases present in up to 16–23% of cases, and even a small CCV case series reports distant spread in a subset of patients [40,41,43]. The absence of distant metastases in our cohort may reflect the small sample size, early-stage detection, or limited follow-up.

Microscopic ETE occurred in 53.3% and gross ETE in 40% of HRV cases, which is significantly higher than the occurrence in cPTC (12.5% for microscopic and 5% for gross ETE, *p* < 0.001). This, too, is consistent with the findings of individual studies for the TCV (53.6% vs. 30.2%, *p* < 0.0001 and 31.42% vs. 5.06% *p* < 0.0001) and is backed by meta-analyses for all aggressive variants, which have shown higher odds of ETE in the TCV (OR = 5.38; 95% CI = 3.76–7.71, *p* < 0.001), DSV (OR = 2.13, 95% CI = 1.74–2.59 and OR = 2.96; 95% CI: 2.04–4.30), and HV (reported in 50–68% of cases) [30,33,43,45,46]. Song et al. also showed ETE to be an independent predictor of poor outcome in multivariate analysis in the CCV [49].

Multifocal tumors were found in 66.7% of HRV cases versus 48.8% in cPTC (*p* < 0.001). This trend, though not as strong as in other characteristics, is still significant. Liu et al. found an OR of 1.34 (95% CI = 1.19–1.51, *p* < 0.001) for multifocality in the TCV compared to cPTC [46]. The DSV, on the other hand, did not show a significantly higher rate of multifocality in meta-analyses [33,47]. Bilateral presentation was seen in 66.7% of HRVs, which is significantly higher than 37.5% for cPTC (*p* < 0.001). Bilaterality is strongly associated with aggressive variants, particularly the DSV and HV, as Poma et al. identified a significantly higher rate of bilateral presentation in the HV with >30% hobnail features [50]. Bilaterality was a significant risk factor for recurrence in the DSV, as shown by Yang et al. [35].

RAI therapy was administered in 84.6% of HRV cases compared to 37.5% of cPTC cases (*p* < 0.001), reflecting more aggressive disease. Multiple studies confirm that HRV patients are more likely to receive RAI, particularly in the TCV (55% vs. 48.8%, *p* = 0.05) [30]. The HV has also shown poorer RAI responsiveness, and, in the CCV, reduced sensitivity to RAI was observed [13,49].

The recurrence rate in HRVs was 53.8%, a stark contrast to 5.3% in cPTC (*p* < 0.001), which reflected the DFS of 53.8% for 5 years and 40.4% for 10 years (vs. 96.4% and 94.5% for cPTC, *p* < 0.001). This aligns with the literature showing recurrence rates of 17.1% in the TCV (vs. 6.3%, *p* = 0.02), 25.7% in the DSV (vs. 5.7%, *p* = 0.003), and up to 36% in the HV [35,43,45]. Malandrino et al. showed the rate of recurrence was significantly higher in patients with the DSV (OR = 3.19, 95% CI = 1.86–5.49), and even higher in those who did not receive RAI treatment postoperatively (OR = 5.09; 95% CI = 2.91–8.90) [47]. Longheu et al. showed a five-year DFS rate of 82.3% in the TCV group (vs. 92.8% in the cPTC group, *p* = 0.0018) [45]. The CCV group also had significantly poorer DFS, with a hazard ratio (HR) of 12.19 (95% CI: 2.11–70.33, *p* = 0.005) [49].

Cancer-specific death occurred in 30% of HRV cases, while no cancer-specific deaths were observed in cPTC (*p* < 0.001). The OS for HRVs was 76.9% at 5 years and 68.4% for 10 years (vs. 98.1% and 96% for cPTC, *p* = 0.001), and the DSS was the same as OS at 5 and 10 years (vs. 100% for both for cPTC, *p* < 0.001). This aligns with survival rates in the literature. For the TCV, the 5 years OS was 69% vs. 94% for other PTCs (*p* < 0.001), and the DSS was 83% vs. 98% at 5 years and 64% vs. 96% at 10 years (*p* < 0.001), with a mean follow-up time for the TCV of 7.7 years vs. 10.9 years for other PTCs [29]. In a meta-analysis by Liu et al., the OR for OS in the TCV was 3.89 (95% CI = 1.93–7.88, *p* < 0.001) and the OR for DSS was 5.86 (95% CI = 3.16–10.87; *p* < 0.001) [46]. There was also a significantly lower OS in the DSV compared to cPTC (OR = 1.89; 95% CI: 1.36–2.62) [33]. Donaldson et al. reported a pooled disease-specific mortality of 10% in the HV [43]. Chen et al. presented nine patients with the CCV (one of whom was diagnosed postmortem) and observed disease-specific death in three patients, two of whom had verified distal metastases, and, at the end of the follow-up, one patient had disease recurrence with verified distal metastases, showcasing how aggressive the CCV can be [41].

The results of our study and its alignment with the literature strongly reinforce and justify the classification of the TCV, DSV, CCV, and HV as high-risk variants. All key clinicopathological features—particularly vascular invasion, ETE, LN metastases, recurrence, and cancer-specific death—occurred at significantly higher rates in HRVs compared to cPTC. It is worth noting that several publications compared the sizes of primary tumor and ages at diagnosis, which we could not apply to our discussion as we matched the patients for age (younger or older than 55 years) and tumor size (1–20 mm, 21–40 mm, 41–60 mm, >60 mm). These findings are consistent with the extensive body of literature, despite the small sample size for the individual variant, supporting the validity of our combined HRV group approach. We further recommend grouping these variants in HRVs due to the rarity of these variants, as the biological and clinical behavior of the TCV, DSV, CCV, and HV, as discussed, are very similar to one another, justifying this sort of unification.

Although preoperative identification of the PTC variant remains challenging, the findings of our study, together with the extensive body of existing literature, support the adoption of a more intensive therapeutic and surveillance strategy for patients with tumors in the HRV subgroup. Specifically, we recommend routine administration of radioactive iodine (RAI) therapy, closer postoperative monitoring with more frequent thyroglobulin measurements and ultrasonographic examinations compared with conventional PTC (cPTC), and less reluctancy for additional surgical intervention. This may include completion (staged) thyroidectomy following initial lobectomy, as well as more extensive (or even prophylactic) central and lateral neck lymph node dissection [51]. In cases of limited RAI-avidity or advanced disease, molecular genetic testing and consideration of targeted therapies are warranted [52]. Nevertheless, clinical decision-making should remain individualized, considering tumor size, patient age, and the full spectrum of clinicopathological characteristics identified on PH examination.

### 4.2. Intermediate-Risk Variant

The SV of PTC was classified into the IRVs for the purpose of this study.

**Figure 8:**
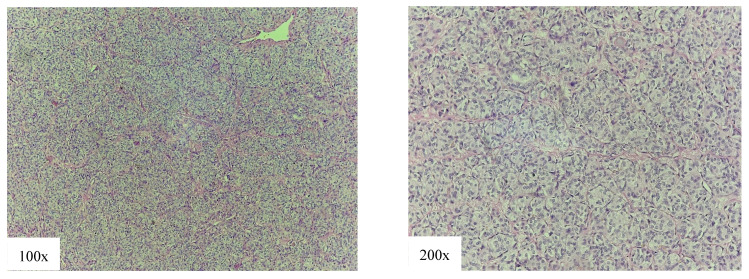
Solid variant of papillary thyroid carcinoma: solid cell nests and trabecules of tumor cells.

The SV represents 2.6–3% of all PTCs [12,53]. Histological features of the SV are solid, trabecular, or nested growth pattern with intervening fibrovascular bands, without tumor necrosis and high mitotic rate. For histological diagnosis to be made, it is necessary for the solid growth pattern to include between 50 and 70% of the tumor mass, while, according to some authors, even 50% is sufficient to diagnose this variant [26]. In our patients, the diagnosis of this subtype is made with less strict criteria of over 50% (Figure 8).

Our findings regarding the SV were favorable and comparable to cPTC, and patients even had a significantly lower chance of having bilateral tumor presentation (Table 2). We had no disease-specific deaths, and recurrence rate was very comparable to cPTC (5-year DFS 96.2% vs. 96.4%, *p* = 0.8) (Table 3).

Although the solid variant of PTC has traditionally been regarded as an aggressive subtype, conflicting evidence has led many to reconsider this classification. A 2018 systematic review and meta-analysis by Vuong et al., which included 205 confirmed SV cases from 11 studies, found no significant difference in the rates of ETE, LN, and distal metastases when compared to cPTC. The risk of vascular invasion was higher (OR = 6.73; 95% CI 3.29–13.78), with a lower DFS and DSS, meaning the recurrence rate was much greater (HR = 3.54; 95% CI 1.32–9.48) and disease-specific death occurred in significantly more patients with the SV (OR = 6.20; 95% CI 2.10–18.30) [4]. However, these results were somewhat biased, as the majority of DSS results were driven by only one study, published over 30 years ago [54]. In contrast to these findings, a group of researchers concluded on the cohort of 20 patients with the SV the same rate of local recurrence compared to cPTC patients (15% vs. 15%), but a higher rate of distant metastases and disease-specific death (10% vs. 0 on both accounts) [53]. In a subsequent study, Chang et al. attributed the aggressive nature of the SV primarily to its locally invasive characteristics, as the rate of ETE was high (50%). The frequency of LN and distant metastases, as well as the rate of local recurrence, was not significantly greater than in cPTC [55]. Adding to these findings, a multi-institutional study by Bin Xu et al. found excellent disease-specific (96%) and disease-free (87%) survival rates among their 156 patients with SV tumors. Based on these findings, they proposed reconsidering the classification of the SV of PTC as an aggressive subtype [6]. Yet, Ivanov et al. provided a contradictory perspective, reporting that the SV was detected in 21% of all lethal PTC cases, compared to only 2% in a control group [56].

What likely explains why earlier studies overestimated the aggressiveness of the SV is the potential for poorly differentiated thyroid carcinoma (PDTC) or other high-grade thyroid carcinomas to have been included in the case groups, as they can also exhibit a solid growth pattern. This is particularly relevant given the recent introduction of differentiated high-grade thyroid carcinoma (DHGTC) in the 2022 WHO histological classification of thyroid neoplasms, which can also be characterized with solid growth patterns besides invasive features, the presence of >3 mitoses per 2 mm^2^, and/or tumor necrosis [5,57]. Consequentially, many authors are beginning to suggest that tumors with solid growth patterns should be reclassified as DHGTC [58]. When considering PDTC, the distinction from the SV should be made following the established Turin criteria—(1) presence of a solid/trabecular/insular growth pattern, (2) absence of the conventional nuclear features of papillary carcinoma, and (3) presence of at least one of the following: convoluted nuclei; >3 mitoses per 2 mm^2^, and tumor necrosis [59,60].

Our findings seem to greatly side with authors disputing the label of the SV as an aggressive subtype, as both the clinicopathological profile and survival rates of the SV were very comparable to cPTC in our cohort.

### 4.3. Low-Risk Variants

Lastly, the third group in our study, LRVs, included the OV and WLV.

**Figure 9:**
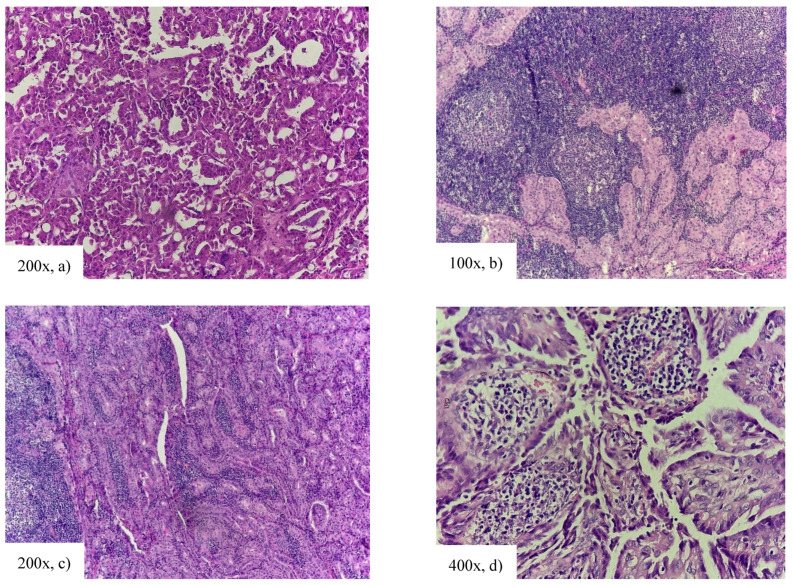
(**a**) Oncocytic variant of papillary thyroid carcinoma: oncocytic cells organized in papillae with intranuclear inclusions; (**b**–**d**) Warthin-like variant of papillary thyroid carcinoma: papillary structure of tumor with high limphoplasmocytic infiltrate within papillae.

The OV has a reported incidence ranging from 1 to 11%, being more common in older female patients [7,61]. Even though the OV is more commonly found in the background of Hashimoto thyroiditis (HT), the subtype that is usually strongly associated with HT is the WLV [10,62], with some studies suggesting up to 80% WLV accompanied by HT [63] (Figure 9).

Investigating the LRV, we did conclude a significantly higher rate for the most clinicopathological characteristics (capsular invasion, vascular invasion, LN metastases, multifocal, and bilateral presentation) in this group compared to cPTC, and more patients with these two subvariants were treated with RAI therapy (Table 2). However, recurrence rate and rate of cancer-specific death was very comparable in both groups, with the same 5- and 10-year DSS. The 5-year DFS was higher in the LRV than cPTC (100% vs. 96.4%) and lower at the 10-year mark (90% vs. 94.5%), but this difference was not statistically significant.

In their cohort including 21 patients, Carr et al. have concluded insignificant differences between the OV and cPTC for ETE (19% vs. 24%, *p* = 0.71), multifocal disease (48% vs. 38%, *p* = 0.53), lymphovascular invasion (9.5% vs. 0, *p* = 0.15), and LN metastases in central (57% vs. 62%, *p* = 0.75) and lateral (19% vs. 24%, *p* = 0.71) compartments, as well as recurrence rate (4.5% for OV vs. 9% for cPTC, *p* = 0.55) [9]. Okuyucu et al. have even compared the OV to both the TCV and cPTC. In the study, the recurrence rate was 16% in the OV, 13.5% in cPTC, and 56% for the TCV, making the recurrence rate of the OV very comparable to cPTC. Recurrence has been found in cPTC more significantly at earlier stages (I and II), while the OV had a higher rate of occurrence in later stages (III and IV) compared to cPTC [11]. Olmos et al. have concluded similar clinical presentation and prognosis between cPTC and the WLV, as rate of multifocality (38% cPTC vs. 59% WLV), rate of ETE (28.4% vs. 17.6% for microscopic and 3.3% vs. 0% for macroscopic), and rate of lymphovascular invasion (21% vs. 12%) were not significantly different between the two groups. cPTC had more patients with a high risk of recurrence (5%) compared to the WLV group (0%), but those results were not statistically significant either. RAI was administered in significantly more patients with cPTC (63% vs. 30%, *p* = 0.017) [10]. Few case reports have reported lymphonodal metastases in the WLV, accentuating how this variant should not be taken lightly, but this should not come as a surprise, as cPTC is also well-known to metastasize in LN [64,65].

Even though the OV and WLV had a higher rate of unfavorable clinicopathological characteristics, the OS, DSS, and DFS were excellent, which in our opinion justifies their placement in the low-risk variant subgroup, despite their clinicopathological profile.

### 4.4. Risk Factors for Disease Recurrence

The univariate analysis we conducted for DFS had identified seven risk factors that can lead to a higher tumor recurrence, namely, vascular invasion, multifocal presentation, bilateral presentation, LN metastases, and microscopic and gross ETE, as well as the classification of tumors in the HRV subgroup. Using multivariate analysis, it was shown that the histological subtype of the tumor alone can greatly impact the rate of recurrence in the high-risk variant group (Table 4). This is in concordance with several previously cited studies reviewing literature for the TCV, DSV, and CCV, which claim that these histological subtypes alone can be a significant prognostic factor for a lower DFS: 81.9% vs. 91.3% for the TCV; OR = 6.288 (95% CI 1.900–20.811, *p* = 0.003) for the DSV; and HR = 5.04 (95% CI: 1.91–13.32, *p* = 0.001) for the CCV [30,37,48]. Other factors which had been shown to affect survival on multivariate analyses were age, tumor size, ETE, LN, and distant metastases [53]. All these factors, and more, significantly impacted the recurrence rate in our study, although only in univariate analysis. Regression analysis for factors impacting DSS could not be properly calculated due to the lack of heterogeneity of data, i.e., all cancer-specific death belonging to the HRV group.

### 4.5. Limitations

The most prominent limitation of this study is the small individual cohort of certain subtypes of PTC, namely, the TCV, DSV, CCV, and HV, which was expected given how rare they are, especially the CCV and HV. This is why we bypassed this limitation by unifying these variants into a larger high-risk variant subgroup which acted as one cohort group of 15 cases. We justify this unification by the extensive body of research previously discussed, which shows that all of these subtypes have similar clinical behavior and prognosis. The new limitation derived from this unification is the inability to reliably draw a conclusion regarding these four subtypes individually.

Secondly, the IRV, LRV, and cPTC (control) groups had far fewer overall and cancer-specific deaths than the HRV subgroup, which is why risk factors for overall death and disease-specific deaths could not be calculated the same way they could be for disease recurrence, due to the lack of heterogeneity of the data.

Lastly, this was a retrospective, single-center study. Despite re-review, residual overlap between aggressive PTC variants and WHO 2022 high-grade categories cannot be entirely excluded and represents an inherent limitation of historical cohorts. Considering how rare these PTC variants are, the study design would greatly benefit from a multicentric approach.

## 5. Conclusions

Due to very unfavorable clinicopathological characteristics and survival rates, the TCV, CCV, DSV, and HV have justified their title as aggressive variants and the subcategorization into the high-risk variant subgroup in this research, with multivariate analysis proving the histological subtype alone can negatively affect DFS. On the other hand, the solid variant of PTC, once thought to be aggressive, has, in the more recent literature, as well as our own study, proved to have comparable clinicopathological characteristics and survival rates to cPTC, and, providing an explanation as to why that might be, we propose the SV not be labeled as an aggressive variant. The OV and WLV might show a statistically higher rate of most unfavorable clinicopathological characteristics, but, with their excellent survival rate, the subcategorization into the low-risk variant is justified and the results were expected.

We recommend this sort of subcategorization for further research. For everyday clinical practice, we recommend that clinicians should pay special attention to tumors from the HRV group (TCV, CCV, DSV, and HV)—RAI therapy, more extensive surgical treatment, more frequent follow-ups, and target therapy when genetic mutations are proven. Caution is still advised for the SV, OV, and WLV, but treatment should not substantially differ from the treatment of cPTC.

## Figures and Tables

**Table 2:** Comparison of clinicopathological features among different groups of rare variant subtypes.

Clinicopathological Features	cPTC	HRV	HRV vs. cPTC	IRV	IRV vs. cPTC	LRV	LRV vs. cPTC
Capsular invasion	26.3%	60%	***p* = 0.015**	15%	*p* = 0.1	64%	***p* = 0.043**
Vascular invasion	5%	53.3%	***p* < 0.001**	2.5%	*p* = 0.67	32%	***p* < 0.001**
LN metastases	5%	40%	***p* < 0.001**	2.5%	*p* = 0.67	28%	***p* = 0.003**
Distant metastases	0%	0%	*p* = 1.0	0%	*p* = 1.0	0%	*p* = 1.0
Microscopic ETE	12.5%	53.3%	***p* < 0.001**	7.5%	*p* = 0.54	20%	*p* = 0.34
Gross ETE	5%	40%	***p* < 0.001**	2.5%	*p* = 0.67	8%	*p* = 0.63
Multifocal presentation	48.8%	66.7%	***p* < 0.001**	20%	*p* = 0.45	52%	***p* < 0.001**
Bilateral presentation	37.5%	66.7%	***p* < 0.001**	10%	*p* < 0.001 *	50%	***p* < 0.001**
Recurrence	5.3%	53.8%	***p* < 0.001**	3.6%	*p* = 1.0	4.3%	*p* = 1.0
RAI treatment	37.5%	84.6%	***p* < 0.001**	24%	*p* = 0.87	60.9%	***p* = 0.043**
Cancer-specific death	0%	30%	***p* < 0.001**	0%	*p* = 1.0	0%	*p* = 1.0

*p*-value was bolded if statistically significant; * high statistical significance achieved in favor of higher incidence in control group; LN—lymphonodal; ETE—extrathyroid extension; RAI—radioactive iodine; cPTC—classic variant; HRV—high-risk variant; IRV—intermediate-risk variant; LRV—low-risk variant

**Table 4:** Univariate and multivariate regression analysis for disease-free survival.

	Univariate			Multivariate		
	HR	95% CI	*p*	HR	95% CI	*p*
Capsular invasion	2.22	0.68–7.25	0.19			
Vascular invasion	5.89	1.67–20.76	0.006	0.19	0.009–4.29	0.29
Multifocal presentation	4.28	1.11–16.45	0.034	0.55	0.003–107.5	0.83
Bilateral presentation	6.38	1.65–24.67	0.007	5.63	0.03–1076	0.52
LN metastases	11.25	3.14–40.32	<0.001	4.1	0.43–39	0.22
Microscopic ETE	9.43	2.72–32.75	<0.001	1.29	0.057–29.2	0.87
Gross ETE	20.0	5.12–78.13	<0.001	6.36	0.31–128.7	0.228
HRV	22.46	4.83–104.34	<0.001	15.82	1.76–142.3	0.014
IRV	0.66	0.07–6.54	0.72			
LRV	1.07	0.1–10.76	0.95			

LN—lymphonodal; ETE—extrathyroid extension; HRV—high-risk variant; IRV—intermediate-risk variant; LRV—low-risk variant; HR—hazard ratio.

## Data Availability

The original contributions presented in this study are included in the article. Further inquiries can be directed to the corresponding authors.
